# Mitochondria-derived vesicles: potential nano-batteries to recharge the cellular powerhouse

**DOI:** 10.20517/evcna.2023.71

**Published:** 2024-06-10

**Authors:** Shalini Mishra, Gagan Deep

**Affiliations:** 1 Department of Internal Medicine-Gerontology and Geriatric Medicine, Wake Forest University School of Medicine, Winston-Salem, NC 27101, USA.; 2 Sticht Center for Healthy Aging and Alzheimer’s Prevention, Wake Forest University School of Medicine, Winston-Salem, NC 27101, USA.; 3 Atrium Health Wake Forest Baptist Comprehensive Cancer Center, Wake Forest University School of Medicine, Winston-Salem, NC 27101, USA.

**Keywords:** Mitochondria, mitochondria-derived vesicle, inter-organelle communication, F1-F0 ATP synthase

## Abstract

Mitochondria dysfunction is increasingly recognized as a critical factor in various pathogenic processes. The mechanism governing mitochondrial quality control serves as an adaptive response, ensuring the preservation of mitochondrial morphology, quantity, and overall function, crucial for cell survival. The generation of mitochondria-derived vesicles (MDVs) is one of the processes of mitochondrial quality control. Recent literature has suggested MDV heterogeneity; however, the detailed characteristics of various MDV subtypes still need to be studied better. Recent studies have shown that MDVs also play a role in inter-organelle communication for mitochondria besides quality control. For instance, Hazan *et al.* demonstrated that functional mitochondria from *Saccharomyces cerevisiae* release vesicles independent of the fission machinery. These vesicles, falling within the typical size range of MDVs, were selectively loaded with mitochondrial proteins, especially with functional ATP synthase subunits. Intriguingly, these MDVs maintained membrane potential and could generate ATP. Moreover, MDVs could fuse with naïve mitochondria, transferring their ATP generation machinery. Lastly, this study revealed a potential delivery mechanism of ATP-producing vesicles, presenting a promising avenue to rejuvenate ATP-deficient mitochondria. Overall, this study unveils a novel mechanism for inter-organelle communication by vesicles, which is crucial for maintaining cellular homeostasis and could also be important in pathological conditions.

Mitochondria, a double membrane-bound organelle, regulate a plethora of cellular functions, encompassing oxidative ATP production, β-oxidation, calcium signaling, and apoptosis. Mitochondria crucially influence energy metabolism, redox status, molecular signaling, and cell survival and death. Owing to their crucial involvement in multiple cellular processes, mitochondrial damage has been considered one of the main driving factors for diseases such as aging-associated pathologies, neurodegenerative diseases, cancer, metabolic diseases, *etc.*^[1–5]^. It is emerging that under both normal physiological conditions and stress situations, mitochondria could communicate with other organelles within the cell and with organelles of other cells. One possible mode of this communication is via mitochondria-derived vesicles (MDVs)^[6–8]^. MDVs are single or double-membrane vesicles generated either from the outer mitochondrial membrane or from the inner and outer mitochondrial membrane with a portion of the mitochondrial matrix, respectively^[9–11]^. MDVs are characterized by these three major criteria: (i) a relatively uniform diameter ranging from 70–150 nm; (ii) biogenesis independent of the mitochondrial fission protein Drp1 (Dnm1 in yeast); and (iii) selective incorporation of protein cargo. While MDV generation is a basal housekeeping process under normal physiological settings, it escalates during pathological circumstances^[7,8,12]^ and is reported to gradually decline with aging^[13]^.

MDVs are highly heterogeneous and their heterogeneity is mainly dependent upon cell type secreting these vesicles and the processes in which these vesicles are involved. Biogenesis of MDVs requires the recruitment of either PINK1/Parkin or Ras-related protein (Rab9) and sorting Nexin 9 (SNX9) or β-barrel protein, mitochondrial-anchored protein ligase (MAPL), and translocase of the outer mitochondrial membrane complex subunit 20 (TOM20)^[6,14–16]^. Initially, MDVs were thought of as vesicles generated in response to oxidative stress as a mitochondrial quality control process where mitochondria package their oxidized cargo in these vesicles and shuttle to the lysosome for degradation, thereby regulating mitochondrial mass more rapidly than mitophagy^[17,18]^. However, recent studies have shown that alteration in MDV generation and release is associated with various pathological conditions such as neurodegeneration, cardiomyocyte damage, accelerated aging, autoimmune diseases, cancer, and infections^[10,19–22]^. During Parkinson’s disease, circulating extracellular vesicles include MDVs, as indicated by the identification of mitochondrial signatures, which could be utilized as biomarkers for disease severity^[23,24]^. On the other hand, Ramirez *et al.* have shown that cannabidiol increases MDV generation by activating mitochondrial permeability transition pore (mPTP) opening^[25]^, thereby advocating that protective MDV formation could potentially serve as a new therapeutic target for neurodegenerative diseases associated with mitochondrial dysfunction. Recently, Li *et al.* showed that MDVs protect cardiomyocytes by delivering Bcl-2 to severely damaged mitochondria under hypoxic stimulation^[12]^. Picca *et al.* showed that in older population with physical frailty and sarcopenia, circulating extracellular vesicles include MDVs, as indicated by the identification of mitochondrial signatures among them, and their secretion reduced despite the increase in the small EV secretion, indicating mitochondrial dysfunction^[26]^. Although, in recent years, our understanding of the potential role of MDVs in normal physiology and under disease conditions has significantly improved, there is still a significant gap in understanding their characteristics and functions, especially their role in inter-organelle communication. Further, given the recent research focus on understanding mitochondrial function in various diseases, there is a pressing need to better comprehend MDVs’ role in maintaining cellular homeostasis. A better understanding of the mechanistic framework of MDV could also aid in developing novel therapeutic approaches for treating various diseases.

A recent study by Hazan *et al.* meticulously characterized MDVs from the isolated functional mitochondria of yeast (strain: *Saccharomyces cerevisiae*)^[27]^. MDVs were isolated by filtration and differential centrifugation and characterized for several known basic characteristics of MDVs^[27]^. It was demonstrated that MDVs were composed of single or potentially double membranes, and their size ranged from 80–200 nm with a peak at 105.8 ± 2.2 nm, corresponding to typical MDV size^[27]^. Further, no change in the MDV secretion profile was observed in Dnm1 knockout cells, confirming that MDV secretion is independent of mitochondrial fission^[27]^. Interestingly, only functional mitochondria were involved in MDV secretion, and over time, as mitochondria lost their ATP generation capacity, MDV concentration decreased and stabilized at a very low level at 48 h^[27]^. This study also identified the selectivity of MDV protein cargo by LC/MS/MS analysis^[27]^. Furthermore, a comparison between mitochondrial and MDVs fractions showed distinct protein distribution between the MDVs and mitochondrial fractions^[27]^. MDVs were more enriched for outer membrane proteins, while mitochondrial ribosomal proteins were relatively less abundant in MDVs compared to their levels in the mitochondrial fractions^[27]^. Additionally, specific enrichment of a few proteins was noted in MDVs^[27]^. For example, isocitrate lyase 1 (Icl-1) was noticeable in MDVs but was present in negligible amounts in the mitochondrial fraction. In short, this study provided compelling evidence for the specific cargo distribution in MDVs. Notably, this study also demonstrated the role of specific cargo proteins in MDV formation. Specifically, malate dehydrogenase 1 (Mdh1), a mitochondrial matrix TCA (tricarboxylic acid) enzyme, and Om45, an outer mitochondrial membrane protein, both were identified in the MDVs, and knockout of these proteins in cells resulted in decreased concentration of MDVs. Overall, this study nicely demonstrated the three major characteristics associated with MDVs, as mentioned above.

Intriguingly, the study also provided *in vitro* evidence about the functional importance of MDVs. Mass spectrometry data suggested the presence of multiple subunits of the F1F0-ATP synthase complex, with 13 out of 17 subunits of F1F0-ATP synthase present in MDVs^[27]^. Furthermore, MDVs showed membrane potential and retained significant ATP production capacity^[27]^. Notably, when incubated with Atp2 (ATP synthase subunit beta) respiratory deficient mitochondria, wild-type MDVs increased ATP production by 560%, while incubation with wild-type mitochondria increased ATP production by 30%. Importantly, this study also provided evidence that MDVs are incorporated into mitochondria following uptake and lend their ATP production machinery to the recipient mitochondria. Notably, a few significant observations in the yeast model were validated in MDVs from isolated mitochondria derived from HEK293 cells. Taken together, this study suggested that MDVs are one of the components of a complex cascade of mitochondrial function, which is not only involved in the quality control of mitochondria but also in the rejuvenation of damaged mitochondria, through the transfer of MDVs between healthy and damaged mitochondria.

Further studies are required to understand the potential of MDVs as a biomarker of pathophysiological diseases where mitochondrial dysfunction is the major driving factor. For clinical translation, reliable methods to isolate MDVs from various biofluids need to be developed further. Until now, the only method to collect pure and specific MDVs has been from isolated mitochondria; however, this approach is not feasible for large clinical setups, especially when dealing with archived biofluid samples. To isolate MDVs from biofluids, a better understanding of unique or specific proteins on the surface of MDVs is requisite.

In conclusion, Hazan *et al.* have presented a thought-provoking approach to better understanding MDVs’ physical characteristics and functional role. Further improving our understanding of the MDV biogenesis biology and their role in various pathological conditions could not only provide us with new prognostic/diagnostic markers but also emerge as a new therapeutic approach, e.g., utilizing MDVs to rejuvenate and restore damaged mitochondria.

## Data Availability

Not applicable.

## References

[1] Chistiakov DA, Sobenin IA, Revin VV, Orekhov AN, Bobryshev YV. Mitochondrial aging and age-related dysfunction of mitochondria. Biomed Res Int 2014;2014:238463. 24818134 10.1155/2014/238463 PMC4003832

[2] Lima T, Li TY, Mottis A, Auwerx J. Pleiotropic effects of mitochondria in aging. Nat Aging 2022;2:199–213. 37118378 10.1038/s43587-022-00191-2

[3] Bhatti JS, Bhatti GK, Reddy PH. Mitochondrial dysfunction and oxidative stress in metabolic disorders - a step towards mitochondria based therapeutic strategies. Biochim Biophys Acta Mol Basis Dis 2017;1863:1066–77. 27836629 10.1016/j.bbadis.2016.11.010 PMC5423868

[4] Javadov S, Kozlov AV, Camara AKS. Mitochondria in health and diseases. Cells 2020;9:1177. 32397376 10.3390/cells9051177 PMC7290976

[5] Klein K, He K, Younes AI, Role of mitochondria in cancer immune evasion and potential therapeutic approaches. Front Immunol 2020;11:573326. 33178201 10.3389/fimmu.2020.573326 PMC7596324

[6] Popov LD. Mitochondrial-derived vesicles: recent insights. J Cell Mol Med 2022;26:3323–8. 35582908 10.1111/jcmm.17391 PMC9189329

[7] Peng T, Xie Y, Sheng H, Wang C, Lian Y, Xie N. Mitochondrial-derived vesicles: gatekeepers of mitochondrial response to oxidative stress. Free Radic Biol Med 2022;188:185–93. 35750270 10.1016/j.freeradbiomed.2022.06.233

[8] Picca A, Guerra F, Calvani R, Mitochondrial-derived vesicles: the good, the bad, and the ugly. Int J Mol Sci 2023;24:13835. 37762138 10.3390/ijms241813835 PMC10531235

[9] Sugiura A, McLelland GL, Fon EA, McBride HM. A new pathway for mitochondrial quality control: mitochondrial-derived vesicles. EMBO J 2014;33:2142–56. 25107473 10.15252/embj.201488104 PMC4282503

[10] Heyn J, Heuschkel MA, Goettsch C. Mitochondrial-derived vesicles-link to extracellular vesicles and implications in cardiovascular disease. Int J Mol Sci 2023;24:2637. 36768960 10.3390/ijms24032637 PMC9917113

[11] Soubannier V, Rippstein P, Kaufman BA, Shoubridge EA, McBride HM. Reconstitution of mitochondria derived vesicle formation demonstrates selective enrichment of oxidized cargo. PloS one 2012;7:e52830. 23300790 10.1371/journal.pone.0052830 PMC3530470

[12] Li B, Zhao H, Wu Y, Mitochondrial-derived vesicles protect cardiomyocytes against hypoxic damage. Front Cell Dev Biol 2020;8:214. 32426351 10.3389/fcell.2020.00214 PMC7212461

[13] Guo Y, Guan T, Yu Q, ALS-linked SOD1 mutations impair mitochondrial-derived vesicle formation and accelerate aging. Redox Biol 2024;69:102972. 38056310 10.1016/j.redox.2023.102972 PMC10746562

[14] König T, Nolte H, Aaltonen MJ, MIROs and DRP1 drive mitochondrial-derived vesicle biogenesis and promote quality control. Nat Cell Biol 2021;23:1271–86. 34873283 10.1038/s41556-021-00798-4

[15] Braschi E, Goyon V, Zunino R, Mohanty A, Xu L, McBride HM. Vps35 mediates vesicle transport between the mitochondria and peroxisomes. Curr Biol 2010;20:1310–5. 20619655 10.1016/j.cub.2010.05.066

[16] Matheoud D, Sugiura A, Bellemare-Pelletier A, Parkinson’s disease-related proteins PINK1 and parkin repress mitochondrial antigen presentation. Cell 2016;166:314–27. 27345367 10.1016/j.cell.2016.05.039

[17] Cadete VJ, Deschênes S, Cuillerier A, Formation of mitochondrial-derived vesicles is an active and physiologically relevant mitochondrial quality control process in the cardiac system. J Physiol 2016;594:5343–62. 27311616 10.1113/JP272703 PMC5023710

[18] Soubannier V, McLelland GL, Zunino R, A vesicular transport pathway shuttles cargo from mitochondria to lysosomes. Curr Biol 2012;22:135–41. 22226745 10.1016/j.cub.2011.11.057

[19] König T, McBride HM. Mitochondrial-derived vesicles in metabolism, disease, and aging. Cell Metab 2024;36:21–35. 38171335 10.1016/j.cmet.2023.11.014

[20] Abuaita BH, Schultz TL, O’Riordan MX. Mitochondria-derived vesicles deliver antimicrobial reactive oxygen species to control phagosome-localized staphylococcus aureus. Cell Host Microbe 2018;24:625–636.e5. 30449314 10.1016/j.chom.2018.10.005 PMC7323595

[21] Picca A, Guerra F, Calvani R, Generation and release of mitochondrial-derived vesicles in health, aging and disease. J Clin Med 2020;9:1440. 32408624 10.3390/jcm9051440 PMC7290979

[22] Gagliardi S, Mitruccio M, Di Corato R, Defects of mitochondria-lysosomes communication induce secretion of mitochondria-derived vesicles and drive chemoresistance in ovarian cancer cells. Cell Commun Signal 2024;22:165. 38448982 10.1186/s12964-024-01507-y PMC10916030

[23] Picca A, Guerra F, Calvani R, Mitochondrial-derived vesicles as candidate biomarkers in Parkinson’s disease: rationale, design and methods of the EXosomes in PArkiNson disease (EXPAND) study. Int J Mol Sci 2019;20:2373. 31091653 10.3390/ijms20102373 PMC6566801

[24] Picca A, Guerra F, Calvani R, Mitochondrial signatures in circulating extracellular vesicles of older adults with parkinson’s disease: results from the EXosomes in PArkiNson’s disease (EXPAND) study. J Clin Med 2020;9:504. 32059608 10.3390/jcm9020504 PMC7074517

[25] Ramirez A, Old W, Selwood DL, Liu X. Cannabidiol activates PINK1-parkin-dependent mitophagy and mitochondrial-derived vesicles. Eur J Cell Biol 2022;101:151185. 34915361 10.1016/j.ejcb.2021.151185 PMC8816654

[26] Picca A, Beli R, Calvani R, Older adults with physical frailty and sarcopenia show increased levels of circulating small extracellular vesicles with a specific mitochondrial signature. Cells 2020;9:973. 32326435 10.3390/cells9040973 PMC7227017

[27] Hazan Ben-Menachem R, Lintzer D, Ziv T, Mitochondrial-derived vesicles retain membrane potential and contain a functional ATP synthase. EMBO Rep 2023;24:e56114. 36929726 10.15252/embr.202256114 PMC10157309

